# Tissue-type plasminogen activator induces conditioned receptive field plasticity in the mouse auditory cortex

**DOI:** 10.1016/j.isci.2023.105947

**Published:** 2023-01-07

**Authors:** Caitlin Smart, Anna Mitchell, Fiona McCutcheon, Robert L. Medcalf, Alexander Thiele

**Affiliations:** 1 Molecular Neurotrauma and Haemostasis, Australian Centre for Blood Diseases, Central Clinical School, Monash University, Melbourne, VIC 3004, Australia; 2 Biosciences Institute, Newcastle University, Newcastle upon Tyne NE1 7RU, UK

**Keywords:** Cell biology, Sensory neuroscience

## Abstract

Tissue-type plasminogen activator (tPA) is a serine protease that is expressed in various compartments in the brain. It is involved in neuronal plasticity, learning and memory, and addiction. We evaluated whether tPA, exogenously applied, could influence neuroplasticity within the mouse auditory cortex. We used a frequency-pairing paradigm to determine whether neuronal best frequencies shift following the pairing protocol. tPA administration significantly affected the best frequency after pairing, whereby this depended on the pairing frequency relative to the best frequency. When the pairing frequency was above the best frequency, tPA caused a best frequency shift away from the conditioned frequency. tPA significantly widened auditory tuning curves. Our data indicate that regional changes in proteolytic activity within the auditory cortex modulate the fine-tuning of auditory neurons, supporting the function of tPA as a modulator of neuronal plasticity.

## Highlights

• Tissue-type plasminogen activator causes auditory cortical plasticity
• Neuronal best frequencies shift away from conditioning tone frequencies
• Auditory spectral tuning curves widen following conditioning tone frequencies

## Graphical abstract

**Figure:**
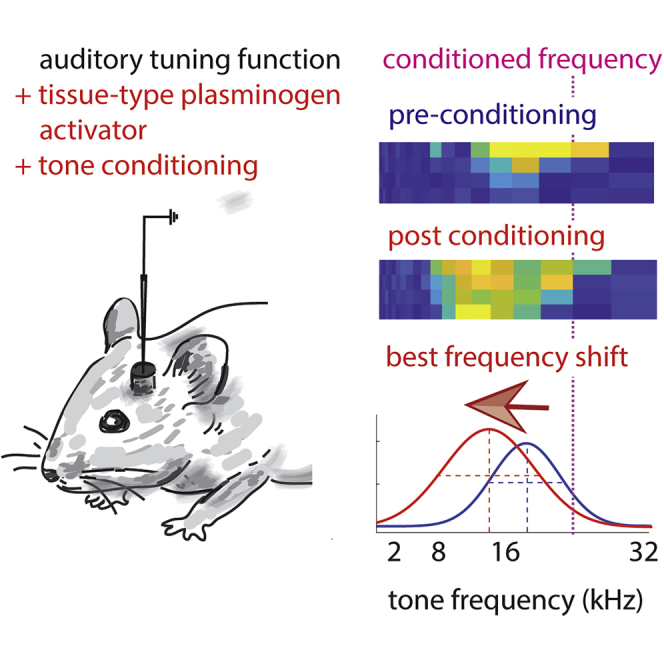

## Introduction

Neuroplasticity is a fundamental capacity of the brain to adapt to developmental or other stimulatory challenges. Neuroplasticity was initially considered to occur primarily during early development being triggered by developmental cues. However, certain aspects of neuroplasticity occur throughout life. Several neurotransmitters and neuromodulators are associated with neuroplasticity, including acetylcholine,^1^^,^^2^^,^^3^^,^^4^^,^^5^^,^^6^^,^^7^ acting through muscarinic^1^^,^^8^^,^^9^^,^^10^ and nicotinic receptors,^11^ glutamate acting via N-methyl-D-aspartate (NMDA)^12^ and α-amino-3-hydroxy-5-methyl-4-isoxazolepropionic acid (AMPA) receptors,^13^ and noradrenaline,^14^^,^^15^^,^^16^ as well as dopamine.^17^^,^^18^

Recently, modulation of synaptic plasticity has additionally been linked with protease activity in the CNS, including matrix metalloproteinases and serine proteases and receptors reviewed by Sonderegger and Matsumoto-Miyai.^19^ These proteases have been proposed to remodel extracellular or perisynaptic protein targets that in turn influence adhesion and reshape the structure of the extracellular matrix and perineuronal nets.^20^

Prominent among the serine proteases associated with plasticity is tissue-type plasminogen activator (tPA), a protease more commonly linked with hemostasis and the removal of blood clots.^21^ However, tPA has a number of important functions in the CNS unrelated to its conventional fibrinolytic capacity.^22^ tPA is expressed in various regions of the CNS including the hippocampus and amygdala.^23^^,^^24^^,^^25^ Non-hemostatic roles for tPA within the CNS include a potential role in synaptic plasticity.^25^^,^^26^^,^^27^ For example, tPA overexpression enhances long-term potentiation (LTP) and enhances learning capabilities in mice. Conversely, tPA inhibition or genetic removal has been linked to diminished or absent LTP.^27^^,^^28^^,^^29^^,^^30^^,^^31^ Mechanistically, tPA modulates NMDA receptor signaling^32^^,^^33^ and the postsynaptic response to glutamate and AMPA receptors in a homeostatic manner.^34^ tPA can also modulate plasticity by processing brain-derived neurotrophic factor (BDNF)^26^^,^^35^ and by signaling via other receptors, including the low-density lipoprotein receptor-related protein (LRP).^30^^,^^31^^,^^32^ However, the role of tPA in neural plasticity at the cellular level *in vivo* has not been investigated. This contrasts with the effects of glutamate, acetylcholine, and noradrenaline. These modulators influence neural plasticity within different cortical areas.^36^ Often, neuronal plasticity is investigated using pairing protocols, where the presence or absence of the drug/modulator of interest determines whether neural tuning functions change after the pairing stimulus has been presented repeatedly.^3^^,^^4^^,^^5^^,^^15^^,^^16^^,^^37^^,^^38^^,^^39^^,^^40^

Here we evaluated the influence of tPA on auditory plasticity in the mouse auditory cortex using previously established pairing protocols.^4^^,^^15^^,^^37^^,^^39^^,^^41^ tPA alters the preferred tuning frequency of neurons. Surprisingly, the effect was dependent on the pairing frequency; if the pairing frequency was above the best frequency (BF), it had a repellent effect, i.e. the BF shifted toward lower frequencies. If the pairing frequency was below the BF, no significant changes occurred. tPA additionally increased the tuning width, i.e. tuning curves were wider following pairing in the presence of tPA. To the best of our knowledge, this is the first report to demonstrate that tPA influences synaptic plasticity and tuning curves in the adult auditory cortex.

## Results

We recorded pre- and post-frequency pairing tuning curves of 44 units from the auditory cortex (A1 based on stereotaxic coordinates) of awake female mice (C57BL6) (across 9 animals) when tPA had been injected locally into the auditory cortex. We compared the effects of frequency pairing in those units to the effects obtained when vehicle (saline) had been injected into the cortex instead (42 units). We included cells into population analysis if the fits of the tuning function to the z-scored data (STAR Methods) for pre- and post-pairing explained at least 70% of the variance. This resulted in inclusion of 37 units when tPA was injected and 29 units when saline was injected.

### Effect of pairing on tuning curves—Unit examples

**Figure 1:**
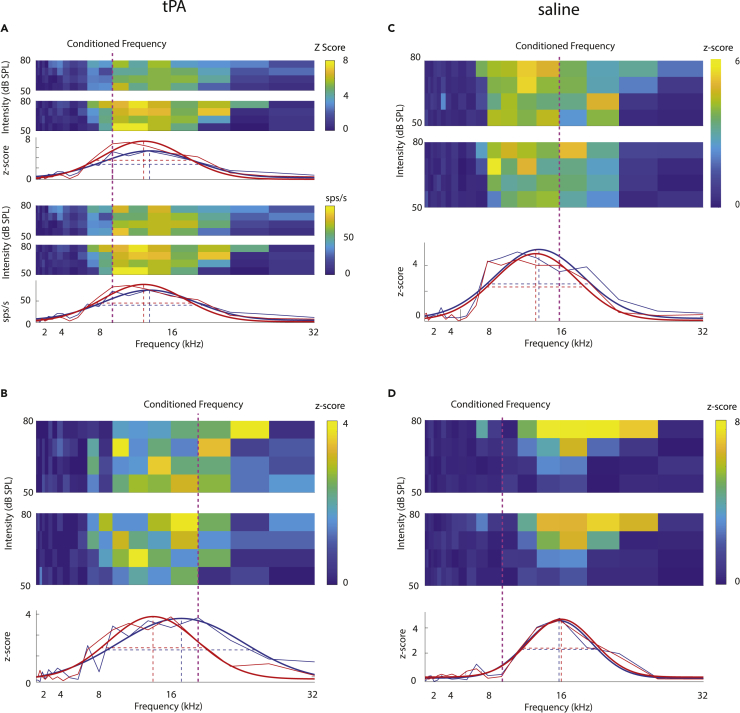
Effect of pairing on tuning curves—unit examples Example frequency tuning curves before and after pairing for units when tPA had been injected into the auditory cortex (A and B) and when saline was injected (C and D). (A) Z-scored (top three panels) and firing rate (lower 3 panels) activity as a function of stimulus frequency (x axis) and intensity (y axis) before pairing was done (pairing frequency: black dashed line). Color-coded surface plots show activity as a function of stimulus frequency (x axis) and stimulus intensity (y axis), before (top surface plots) and after pairing (lower surface plots). Average activity (solid thin lines) across the 4 stimulus intensities is shown in the panels below the surface plots. Activity before pairing is shown in blue and after pairing is shown in red. Fitted tuning functions (solid thick lines) delineate the best frequency (dashed vertical lines) and the tuning width (horizontal dashed lines). The frequency of the conditioning stimulus was below the best frequency (BF) for this unit. (B) Example unit that shows a shift away from the conditioned tuning curve after pairing, when tPA had been injected. For simplicity, only z-scored activity is shown, but results are the same when raw firing rates are used instead. (C) Example unit when saline was injected and the frequency of the conditioning stimulus was below the BF. (D) Example unit when saline was injected and the frequency of the conditioning stimulus was above the BF.

Example tuning curves for 4 different units when tPA (Figures 1A and 1B) and when saline (Figures 1C and 1D) had been injected prior to the experiment are shown in Figure 1. The top part of Figure 1A shows pre- and post-pairing z-scored activity maps and the averaged z-scored activities (averaged across the 4 stimulus intensities) along with fitted tuning curves. The bottom part of Figure 1A shows pre- and post-pairing raw firing rate (spikes/second) maps and the averaged firing rates activities along with fitted tuning curves. The unit had a BF of ∼11 kHz before pairing. The pairing frequency used was 9.51 kHz (presented at a sound pressure level [SPL] of 80 decibels [dB]). After pairing, the BF was largely unchanged. Figure 1B shows an example unit that had a BF of ∼16 kHz before pairing. The pairing frequency used was 19.02 kHz. After pairing, the BF was shifted toward ∼14 kHz, away from the previous BF and away from the pairing frequency, i.e. a repulsive effect occurred when pairing above the BF in the presence of tPA was performed. Figure 1C shows an example of a unit with a BF ∼13 kHz, which remained largely unchanged when saline had been injected and pairing was done with a stimulus of 16 kHz. An example unit when saline was injected and the pairing frequency was below the units BF is shown in Figure 1D. The unit had a BF ∼16 kHz, which remained unchanged after pairing.

Overall, close to identical estimates of BF and tuning width across our dataset were obtained irrespective of whether firing rates (spikes/sec), normalized firing rates, or z-scored data were used. However, offset and gain naturally differed for these different measures as the ranges of input data were different. To allow for better comparison of changes in gain and offset between neurons, we will mostly report the data fitted using z-scored firing rates in the remainder of the paper, but the statistical outcomes for the main parameters of interest (tuning width and BF of neurons) did not differ irrespective of which data format was used for the fitting.

To quantify how drug application and pairing affected the neural tuning, we determined tuning parameters from the fitted Gaussian functions (STAR Methods) under conditions when saline was injected and when tPA was injected at the start of the experiments. To take into account the possibility that pairing differently affected tuning, dependent on whether pairing was done below the BF or above the BF, we separated data according to where the pairing frequency was located relative to the BF. For each parameter of the tuning function (offset, gain, full width at half maximum [FWHM], and location of BF), we then determined whether it was affected by drug (saline/tPA) or by pairing type (pairing frequency above or below the BF) and whether there was an interaction using a mixed-model ANOVA (where before and after pairing were repeated factors, while drug and pairing type were non-repeated). In the following, we will describe effects for the different tuning parameters in succession.

### Effect of pairing on tuning offset

**Figure 2:**
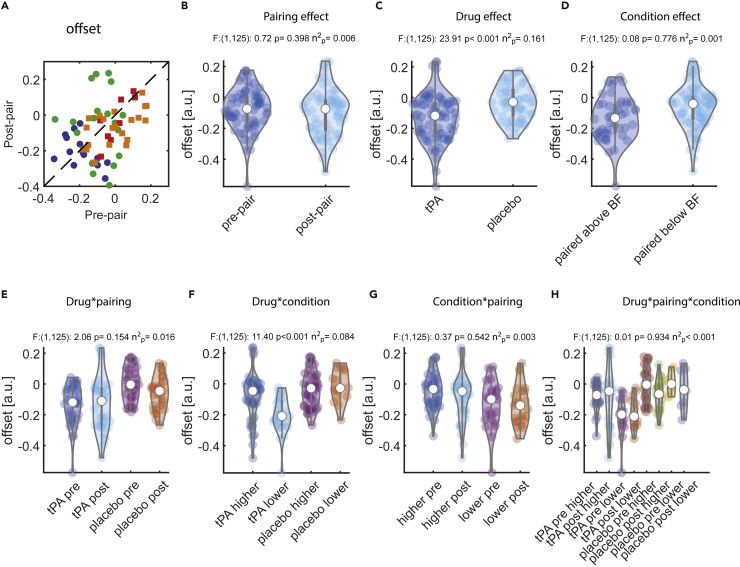
Effect of pairing on tuning offset (A) Green: Pre- and post-pairing offset for units recorded under tPA and when the pairing frequency was above the best frequency. Blue: units recorded under tPA and when the pairing frequency was below the best frequency. Orange: units recorded under saline (placebo) and when the pairing frequency was above the best frequency. Red: units recorded under saline and when the pairing frequency was below the best frequency. (B) Distribution of tuning offset for pre- vs. post-pairing (across all other conditions). (C) Distribution of tuning offset for tPA- vs. saline-treated units (across all other conditions). (D) Distribution of tuning offset for units conditioned with a stimulus above the best frequency vs. a stimulus below the best frequency (across all other conditions). (E) Interaction effect between drug (tPA/saline) and pairing (pre-/post-pairing), pooled across pairing conditions (above/below best frequency). (F) Interaction effect between drug (tPA/saline) and pairing condition (above/below best frequency) pooled across pairing effects (pre-/post-pairing). (G) Interaction effect between pairing (pre-/post-pairing) and pairing frequency (above/below best frequency) pooled across drug conditions (tPA/saline). (H) Triple interaction between drug (tPA/saline), pairing (pre-/post-pairing), and pairing frequency (above/below best frequency). Values above each graph show F-values, p values, and effect sizes calculated from a mixed-model ANOVA.

The pre- and post-pairing offsets for the individual units are shown in Figure 2A. Comparing tuning function offset between pre- and post-pairing did not reveal any significant differences (Figure 2B). This means that the activity to tones that were (relatively) far away from the BF was not affected by the conditioning (no difference between pre- and post-conditioning). However, when tPA was applied, activity to tones that were (relatively) far away from BF was overall lower than when saline was applied, evident by the fact that there was a main effect of drug application on tuning offset (Figure 2C). There was no difference between tuning offset for conditioning frequencies that were above vs. below the BF (Figure 2D). Tuning offset was not affected by drug type (saline/tPA) or pairing (pre-/post-pairing, Figure 2E). A significant interaction of drug type (saline/tPA) and condition type (paired above/paired below the BF) occurred (Figure 2F), whereby responses to tones that were (relatively) far away from the BF were selectively lower after pairing with frequencies below the BF when tPA had been injected (relative to pairing with frequencies above the BF and relative to saline conditions). Offsets differed significantly only for units following tPA (not saline) application. Here, units conditioned with stimuli below their BF overall had lower offsets than units conditioned with stimuli above their BF (t(1,72):3.690, p < 0.001, diff:0.122, CI:0.056 0.188, D'':0.874, post-hoc t-test). No difference was found for units when saline was injected (t(1,56):-0.720, p: 0.475, diff:-0.024, CI:-0.090 0.042, D'':-0.211, post-hoc t-test). We found no interaction of pairing frequency (above/below BF) and pairing itself (pre-/post-pairing, Figure 2G) and also no triple interaction between drug ∗ pairing frequency ∗ pairing (Figure 2H).

### Effect of pairing on tuning width (FWHM)

**Figure 3:**
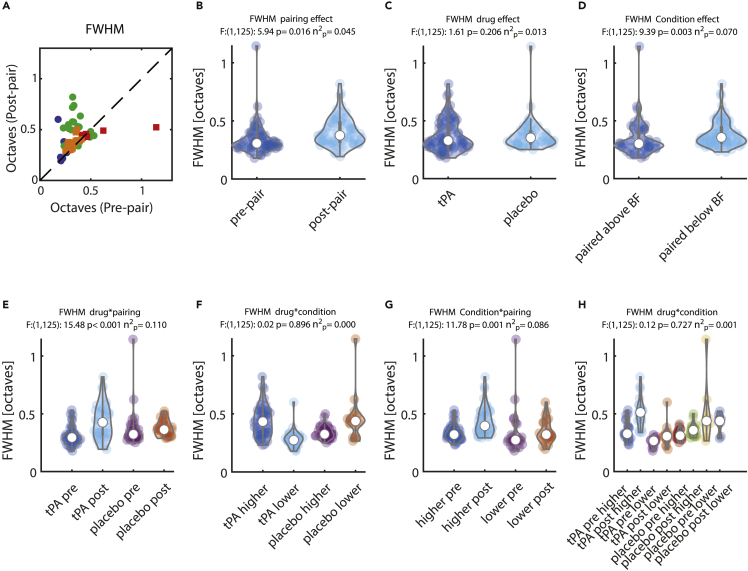
Effect of pairing on tuning width Tuning width is expressed as full width at half maximum (FWHM) in octaves relative to the best frequency. (A) Green: Pre- and post-pairing FWHM for units recorded under tPA and when the pairing frequency was above the best frequency. Blue: units recorded under tPA and when the pairing frequency was below the best frequency. Orange: units recorded under saline and when the pairing frequency was above the best frequency. Red: units recorded under saline and when the pairing frequency was below the best frequency. (B) Distribution of FWHM for pre- vs. post-pairing (across all other conditions). (C) Distribution of FWHM for tPA- vs. saline-treated units (across all other conditions). (D) Distribution of FWHM for units paired with a stimulus above the best frequency vs. a stimulus below the best frequency (across all other conditions). (E) Interaction effect between drug (tPA/saline) and pairing (pre-/post-pairing), pooled across pairing frequencies (above/below best frequency). (F) Interaction effect between drug (tPA/saline) and pairing frequency (above/below best frequency) pooled across pairing effects (pre-/post-pairing). (G) Interaction effect between pairing (pre-/post-pairing) and pairing frequency (above/below best frequency) pooled across drug conditions (tPA/saline). (H) Triple interaction between drug (tPA/saline), pairing (pre-/post-pairing), and pairing frequency (above/below best frequency). Values above each graph show F-values, p values, and effect sizes calculated from a mixed-model ANOVA.

The pre- and post-pairing FWHMs (expressed in octaves of BF) for the individual units are shown in Figure 3A. Our main finding was that tuning width in the presence of tPA was predominantly increased after pairing with a frequency that was higher than the BF (Figures 3A and 3H). In addition, paring in itself (irrespective of which drug or where pairing frequency was located relative to the BF) resulted in a significant increase in tuning width (Figure 3B). There was a no main effect of drug application on tuning width (Figure 3C). Tuning was narrower when paired with stimuli that were above the BF (Figure 3D). FWHM showed a drug type (saline/tPA) ∗ pairing interaction (pre-/post-pairing, Figure 3E). FWHM increased after pairing when units were under tPA influence (t(1,36):-4.827, p < 0.001, n = 37, diff:-0.119, CI:-0.170 -0.069, D'':-0.794, post-hoc t-test) but not when units were under saline influence (t(1,28):-0.141, p: 0.889, n = 29, diff:-0.003, CI:-0.053 0.046, D'':-0.026, post-hoc t-test, Figure 3E). The change in FWHM for pre- vs. post-pairing differed significantly between the drug groups (t(1,64):-3.294, p = 0.002, diff:-0.116, CI:-0.186 -0.046, D'':-0.817, post-hoc t-test). No significant interaction of drug type (saline/tPA) ∗ pairing frequency (paired above/paired below the BF) occurred (Figure 3F). No difference was found for units when saline was injected (t(1,56):-0.720, p = 0.475, diff = −0.024, CI = −0.090 0.042, D' = -0.211, post-hoc t-test). A significant interaction of pairing frequency (above/below BF) and pairing itself (pre-/post-pairing) on FWHM occurred (Figure 3G). Post-hoc testing revealed that tuning width was larger post-pairing if pairing was done with stimuli above the BF (t(1,42):-5.016, p < 0.001, n = 37, diff:-0.102, CI:-0.143 -0.061, D':-0.765, post-hoc t-test) but did not change between pre- and post-pairing when pairing was done with stimuli lower than the BF (t(1,22):-0.152, p: 0.880, n = 29, diff:-0.005, CI:-0.078 0.067, D':-0.032, post-hoc t-test). The change in FWHM upon pairing differed significantly between the two groups (t(1,64):-2.564, p: 0.013, diff:-0.097, CI:-0.172 -0.021, D':-0.662, post-hoc t-test). There was no triple interaction between drug ∗ pairing frequency ∗ pairing (Figure 3H).

### Effect of pairing on tuning gain

**Figure 4:**
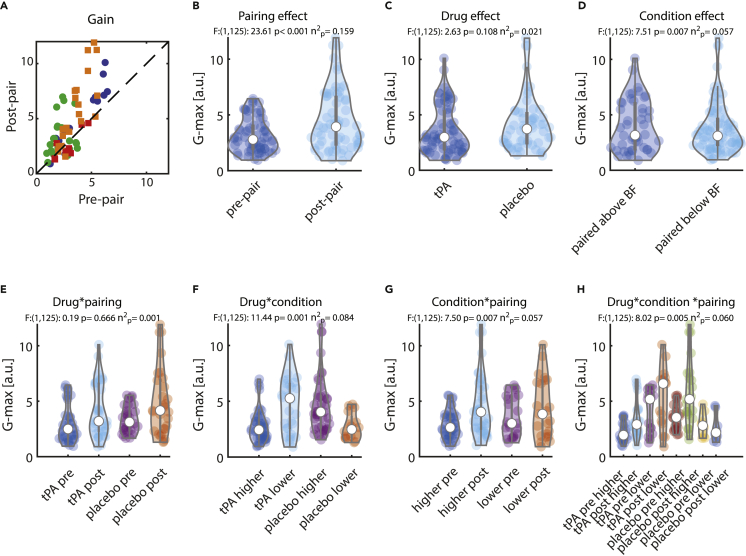
Effect of pairing on tuning gain (height of tuning curve relative to offset) (A) Green: Pre- and post-pairing tuning gain for units recorded under tPA and when the pairing frequency was above the best frequency. Blue: units recorded under tPA and when the pairing frequency was below the best frequency. Orange: units recorded under saline and when the pairing frequency was above the best frequency. Red: units recorded under saline and when the pairing frequency was below the best frequency. (B) Distribution of tuning gain for pre- vs. post-pairing (across all other conditions). (C) Distribution of tuning gain for tPA- vs. saline-treated units (across all other conditions). (D) Distribution of tuning gain for units conditioned with a stimulus above the best frequency vs. a stimulus below the best frequency (across all other conditions). (E) Interaction effect between drug (tPA/saline) and pairing (pre-/post-pairing), pooled across pairing frequencies (above/below best frequency). (F) Interaction effect between drug (tPA/saline) and pairing frequencies (above/below best frequency) pooled across pairing effects (pre-/post-pairing). (G) Interaction effect between pairing (pre-/post-pairing) and pairing frequency (above/below best frequency) pooled across drug conditions (tPA/saline). (H) Triple interaction between drug (tPA/saline), pairing (pre-/post-pairing), and pairing frequency (above/below best frequency). Values above each graph show F-values, p values, and effect sizes calculated from a mixed-model ANOVA.

Tuning gain gives an indication of the differential activity to stimuli far away from the BF and stimuli close to the BF. The pre- and post-pairing gains for the individual units are shown in Figure 4A. Overall this difference increased after pairing, for both tPA and saline conditions, evident by the fact that gain was significantly larger after pairing than before (pooled across all other conditions, Figure 4B). There was no main effect of drug application on tuning gain (Figure 4C). There was a main effect of pairing frequency (paired with a stimulus above vs. below the BF) on gain, whereby gain was smaller when paired with stimuli that were above the BF (Figure 4D), but this was largely driven by increased gains when tPA had been applied and pairing was done with stimuli below the BF (Figure 4F, details below). Gain did not show a drug type (saline/tPA) ∗ pairing interaction (pre-/post-pairing, Figure 4E)). A significant interaction of drug type (saline/tPA) ∗ condition type (paired above/paired below the BF) occurred (Figure 4F). Gain was higher in units treated with tPA if conditioning stimuli were below the BF than above the BF (t(1,72):-4.089, p: 0.000, diff:-1.877, CI:-2.792 -0.962, D':-0.968, post-hoc t-test). Conversely, gain was lower in units treated with saline if conditioning stimuli were above the BF than below the BF (t(1,56):2.955, p: 0.005, diff:1.992, CI:0.642 3.343, D':0.868, post-hoc t-test). A significant interaction of pairing frequency (above/below BF) and pairing itself (pre-/post-pairing) on gain occurred (Figure 4G) for the drug and for the saline condition, whereby post-pairing the gain was increased in both conditions (drug: t(1,42):-5.390, p < 0.00, diff:-1.696, CI:-2.331 -1.061, D':-0.822; saline: t(1,22):-2.763, p: 0.011, n(b) = 29, diff:-0.701, CI:-1.226 -0.175, D':-0.576, post-hoc t-test). Comparing drug and saline condition showed that the effect was larger in the drug (tPA) condition (t(1,64):-2.121, p: 0.038, diff:-0.996, CI:-1.934 -0.058, D':-0.548, post-hoc t-test). We found a triple interaction for tuning gain between drug∗pairing frequency∗pairing (Figure 4H). The statistical details of the triple interaction (post-hoc testing) are shown in Table 1. Tuning gain statistical outcomes were the only ones where substantial differences arose depending on which data were used for the fitting procedures. If raw or normalized firing rates were used for fitting (instead of z-scored data), the main effect of an increase in tuning gain, the condition∗pairing, and the drug∗condition∗pairing effects were not significant. This suggests that pairing and tPA affected (increased) the signal-to-noise ratio of neuronal responses, which are captured by z-scored data but not (or less so) by raw and normalized firing rates.

**Table 1:** Statistical effects of drug and pairing on tuning gain

Group number	Group name	t-statistic	p value	Difference	CI	Effect size (D′)
1	tPA _pre-higher pair_ vs. tPA _post-higher pair_	t(1,42):-2.835	0.007	−1.169	−2.001 -0.337	−0.855
2	tPA _pre-lower pair_ vs. tPA _post-lower pair_	t(1,28):-1.362	0.184	−1.211	−2.992, 0.451	−0.533
3	Saline _pre-higher pair_ vs. Saline _post-higher pair_	t(1,40):-3.100	0.004	−2.249	−3.715, −0.782	−0.957
4	Saline _pre-lower pair_ vs. Saline _post-lower pair_	(1,14):0.425	0.677	0.257	−1.041, 1.556	0.212

Group differences (pre- vs. post-pairing)	t-statistic	p value	Difference	CI	Effect size (D′)
Group 1 vs. 2	t(1,35):0.082	0.935	0.043	−1.008 1.093	0.429
Group 1 vs. 3	t(1,41):1.757	0.086	1.080	−0.161 2.321	0.538
Group 1 vs. 4	(1,28):−2.209	0.036	1.426	2.749 -0.104	−0.912
Group 2 vs. 3	t(1,34):1.639	0.110	1.037	−0.249 2.323	0.554
Group 2 vs. 4	t(1,21):−3.333	0.003	−1.469	−2.385 -;0.552	−1.459
Group 3 vs. 4	t(1,27):−3.074	0.005	−2.506	−4.179, -0.833	−1.277

Post-hoc comparison statistics for the triple interaction found for tuning gain. Within-group comparisons between pre- and post-pairing (repeated measures) are shown in the top 4 rows. Between-group comparisons are shown in the rows thereafter. Here within-group differences were calculated for pre- and post-pairing, and these differences were then compared between groups.

### Effect of pairing on neuronal BF

**Figure 5:**
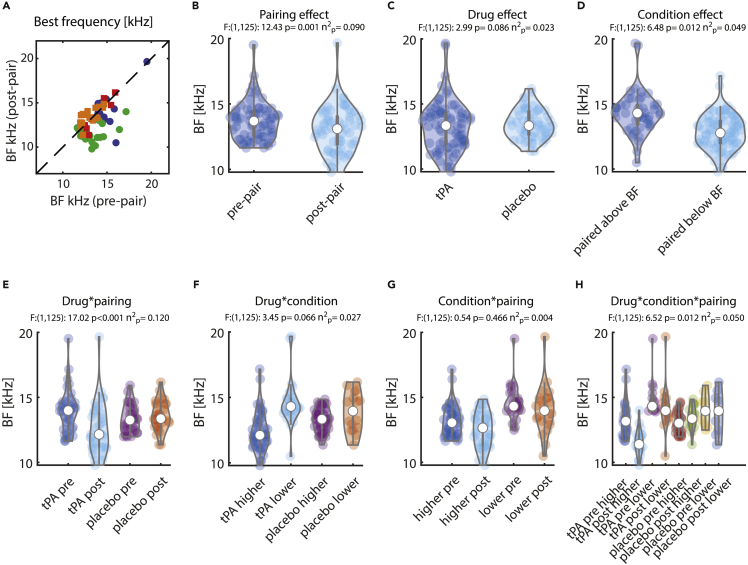
Effect of pairing on best frequency (BF) (A) Green: Pre- and post-pairing BF for units recorded under tPA and when the pairing frequency was above the BF. Blue: units recorded under tPA and when the pairing frequency was below the BF. Orange: units recorded under saline and when the pairing frequency was above the BF. Red: units recorded under saline and when the pairing frequency was below the BF. (B) Distribution of BF for pre- vs. post-pairing (across all other conditions). (C) Distribution of BF for tPA- vs. saline-treated units (across all other conditions). (D) Distribution of BF for units conditioned with a stimulus above the BF vs. a stimulus below the BF (across all other conditions). (E) Interaction effect between drug (tPA/saline) and pairing (pre-/post-pairing), pooled across pairing conditions (above/below BF). (F) Interaction effect between drug (tPA/saline) and pairing condition (above/below BF) pooled across pairing effects (pre-/post-pairing). (G) Interaction effect between pairing (pre-/post-pairing) and pairing condition (above/below BF) pooled across drug conditions (tPA/saline). (H) Triple interaction between drug (tPA/saline), pairing (pre-/post-pairing), and condition stimuli (above/below BF). Values above each graph show F-values, p values, and effect sizes calculated from a mixed-model ANOVA.

One of the main questions in relation to previous pairing studies was whether BFs shift systematically after pairing and whether this was more pronounced when tPA was applied. In summary, we found indeed larger changes in BF after pairing when tPA was applied than when saline was applied, but this depended on whether pairing frequency was above or below the neurons BF. Here, we found a shift away from the pairing frequency under tPA when the pairing frequency was above the BF, but no systematic changes occurred when the pairing frequency was below the BF. The pre- and post-pairing BFs for the individual units are shown in Figure 5A. The details of the statistical assessment are outlined below. BF was significantly lower after pairing than before (pooled across all other conditions, Figure 5B). There was a main effect of drug application on BF, whereby BF was significantly lower under tPA (Figure 5C). There was a main effect of pairing frequency (paired with a stimulus above vs. below the BF) on BF, whereby BF was higher when paired with stimuli that were above the BF (Figure 5D). BF did show a drug type (saline/tPA) ∗ pairing interaction (pre-/post-pairing, Figure 5E). BF was significantly smaller after pairing when tPA was applied (t(1,36):5.046, p < 0.001, diff:1.347, CI:0.805 1.888, D':0.830, post-hoc t-test) but not when saline was applied (t(1,28):-1.661, p = 0.108, diff:-0.230, CI:-0.515 0.054, D'':-0.308, post-hoc t-test). The difference in BF change between the two groups was significant (t(1,64):4.839, p < 0.001, diff:1.577, CI:0.926 2.228, D':1.200, post-hoc t-test). No significant interaction of drug type (saline/tPA) ∗ pairing frequency (paired above/paired below the BF) occurred (Figure 5F). No significant interaction of pairing frequency (above/below BF) and pairing itself (pre-/post-pairing) on BF occurred (Figure 5G). We found a triple interaction for BF between drug ∗ pairing frequency ∗ pairing (Figure 5H). The statistical details of the triple interaction (post-hoc testing) are shown in Table 2. Specifically, reductions in BF after pairing were restricted to units in the tPA group that were paired with stimuli higher than the BFs. However, the modest apparent reduction in BF in the tPA group paired with lower frequencies was also significantly different from the changes in BF that occurred in the saline group paired with higher frequencies, as here the trend was to shift BFs closer to the pairing frequency. These effects did not depend on whether the averaged z-scored data or the averaged normalized firing rates were used for fitting the tuning function.

**Table 2:** Statistical effects of drug and pairing on best frequency

Group number	Group name	t-statistic	p value	Difference	CI	Effect size (D′)
1	tPA _pre-higher pair_ vs. tPA _post-higher pair_	t(1,42):4.751	<0.001	1.780	1.024 2.536	1.432
2	tPA _pre-lower pair_ vs. tPA _post-lower pair_	t(1,28):1.154	0.258	0.711	−0.551 1.972	0.421
3	Saline _pre-higher pair_ vs. Saline _post-higher pair_	t(1,40):-1.462	0.152	−0.394	−0.939, 0.151	−0.451
4	Saline _pre-lower pair_ vs. Saline _post-lower pair_	t(1,14):0.255	0.802	0.199	−1.475 1.873	0.128

Group differences (pre- vs. post-pairing)	t-statistic	p value	Difference	CI	Effect size (D′)
Group 1 vs. 2	t(1,35):2.053	0.048	1.069	0.012 2.127	0.688
Group 1 vs. 3	t(1,41):6.045	<0.001	2.174	1.448 2.901	1.844
Group 1 vs. 4	t(1,28):2.829	0.009	1.581	0.436 2.726	1.168
Group 2 vs. 3	t(1,34):2.781	0.009	1.105	0.297 1.912	0.940
Group 2 vs. 4	t(1,21):0.834	0.414	0.512	−0.765 1.788	0.365
Group 3 vs. 4	t(1,27):−2.012	0.054	−0.593	−1.198 0.012	−0.836

Post-hoc comparison statistics for the triple interaction found for best frequency change. Within-group comparisons between pre- and post-pairing (repeated measures) are shown in the top 4 rows. Between-group comparisons are shown in the rows thereafter. Here within-group differences were calculated for pre- and post-pairing, and these differences were then compared between groups.

Note that all the above analyses were done based on units where the Gaussian fit explained ≥70% of the variance. However, the effects reported in Figures 2, 3, 4, and 5 did not depend on goodness of fit inclusion criteria. If we performed the analysis on our entire cell sample, we obtained qualitatively identical results for almost all significant effects reported. Critically, the results reported in relation to changes in BF and FWHM were unaffected.

### Population tuning curves

**Figure 6:**
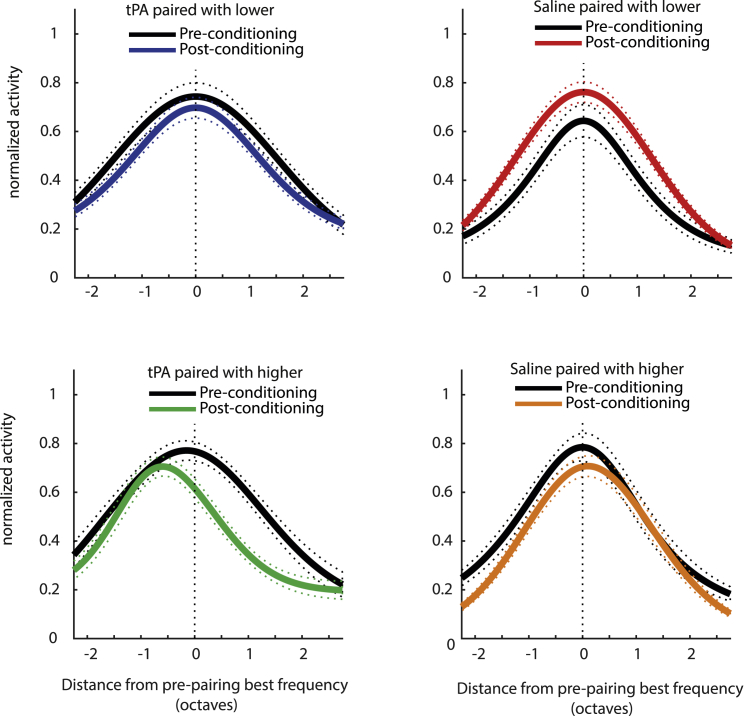
Effect of pairing on fitted population tuning curves Black solid lines show averaged (mean) tuning functions before pairing, aligned to the BF before pairing and set to zero. Colored solid lines show average (mean) tuning functions relative to pre-pairing functions, whereby best frequency distance is expressed in octaves of BF. BFs were systematically shifted toward lower frequencies post-pairing for the sample affected by tPA, provided the conditioning frequency was higher than the BF. A small trend toward increased BFs for the sample affected by saline and conditioned with frequencies above the BF toward upwards shifts is apparent. Pairing reduced tuning gain in the tPA group and increased it in the saline group. Dashed lines show SEM.

To further visualize the effect of tPA application of pairing on BF changes, we determined the BF from each Gaussian tuning function pre-pairing (and plotted the average tuning function aligned to the BF location, Figure 6) and then plotted the population tuning function post-pairing aligned to the pre-pairing BF location for the two tPA conditions and the two saline conditions. The results for normalized activity ranges are shown in Figure 6, but the results on the shifts of tuning functions were robust for raw firing rates and z-scored activity levels.

**Figure 7:**
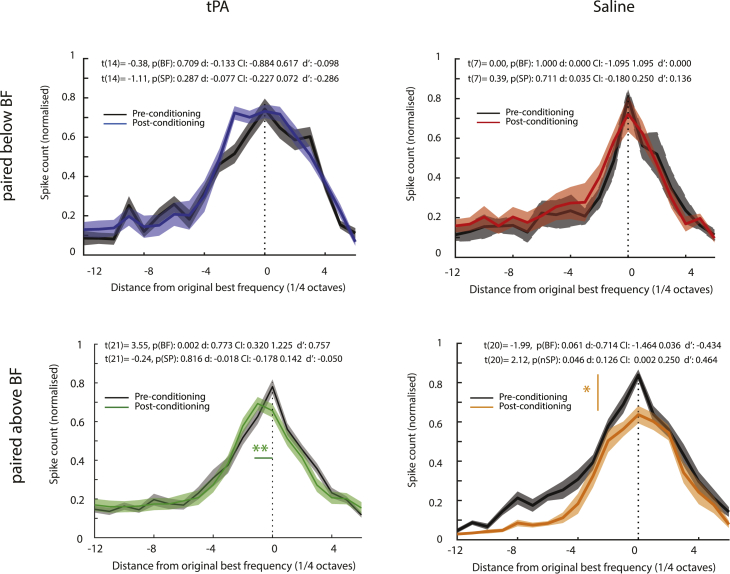
Effect of pairing on non-fitted population tuning curves Black solid lines show averaged tuning functions before pairing, aligned to the BF before pairing and set to zero. Colored solid lines show average tuning functions relative to pre-pairing functions, whereby BF distance is expressed in quarter octave steps relative to BF. BFs were systematically shifted toward lower frequencies post-pairing for the sample affected by tPA, provided the conditioning frequency was higher than the BF. A trend toward increased BFs for the sample affected by saline and conditioned with frequencies above the BF toward upwards shifts is apparent (see inset statistics). Pairing reduced normalized spike counts in the saline group. Solid lines show means, and shaded areas show SEM. Insets show t-test statistics for BF comparison (p(BF)) and for normalized peak spike count (comparison (p(nSP)). d indicates mean difference between groups, CI gives the confidence interval, and d' indicates effect size. Colored lines associated with stars highlight effects that were significant.

To further test whether the change in BF as a function of drug and condition type was an artifact of fitting the tuning function to the data, we determined the pre-pairing BF based on the stimulus frequency that yielded maximal average normalized response (unfitted). These BFs were aligned and set as zero. We then determined the BF post-tuning the same way and plotted these relative to the BF pre-pairing (Figure 7). We performed paired t-tests to determine whether BFs and normalized spiking amplitude (unfitted) at BF significantly differed post-pairing for the four groups. BF was significantly lower after pairing in the tPA-treated group when the conditioning frequency was above the pre-pairing BF (Figure 7, for details about the statistics, see figure inset). There was an opposite trend of significantly *higher* BFs after pairing in the saline-treated group when the conditioning frequency was above the pre-pairing BF (Figure 7, for details about the statistics, see figure inset). The latter group showed significantly reduced tuning amplitude after pairing (Figure 7, for details about the statistics, see figure inset). None of the other groups showed significant differences. Note that the latter effect is different from what was seen for the z-scored fitted functions, where amplitudes were larger after pairing for this group. This difference can arise because the normalized tuning amplitude does not take the spontaneous activity into account, while z-scored data do. If pairing reduces spontaneous activity (and associated SD), then z-scores can increase even if absolute spike counts decrease.

Overall, these results corroborate the finding that BFs in tPA-exposed units shift away from the conditioning frequency if the latter is above the pre-pairing BF. This is opposite to the trend seen in saline-exposed units.

Finally, it could be argued that for the latter analysis, there is no need to select units based on the quality of fit as data extraction is not based on fitted values. If we perform the same analysis without excluding any units, we still find that that pairing above the BF in units exposed to tPA resulted in a shift toward lower BFs (t(27) = 4.36, p(BF) < 0.001, d: 0.964, CI: 0.511 1.418, d': 0.824). For the entire sample of saline-exposed units conditioned with frequencies above their BF, we now find a significant shift toward higher frequencies, not just a trend as with the reduced sample (t(26) = −2.22, p(BF): 0.035, d: −0.630, CI: −1.212 -0.048, d': −0.428). Thus, the effects described are not restricted to units well-fit by a Gaussian tuning curve.

### Location and spread of the injected tPA in the mouse auditory cortex

**Figure 8:**
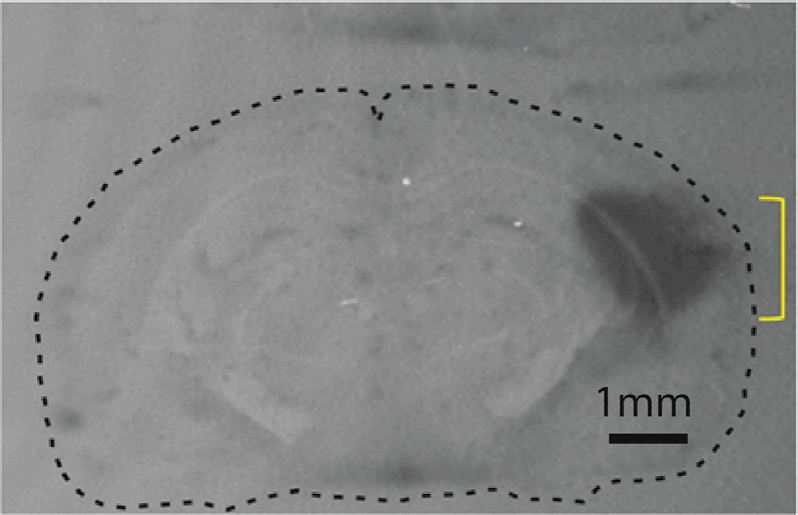
Location and spread of tPA after injection *In situ* zymography was used to visualize the location of fibrinolytic activity after injection of tPA into the auditory cortex (bregma −2.8 posterior; 4.1 mm lateral, depth 0.5 to 1.5 mm). After injection, mice were transcardially perfused with PBS and the brain frozen. Coronal sections (100 μm) were prepared covering the injected area. An agarose matrix containing casein (a plasmin substrate) and plasminogen was then overlaid on the section and left to incubate at 37°C for up to 6 h. The darkened area indicated by the yellow bracket indicates the extent of localized activity due to tPA injection. The black dotted line indicates the external boundary of the coronal section.

To validate that the injected tPA had entered the region of interest, *in situ* zymography was performed on coronal sections of a representative mouse brain following transcardial perfusion to remove blood. As shown in Figure 8, tPA activity was detected within the auditory cortex and was functionally active.

## Discussion

We studied whether tPA application to the auditory cortex of mice affects spectral tuning curves when used in combination with established auditory tone-pairing protocols.^4^^,^^15^^,^^37^^,^^39^^,^^41^ We found that tPA alters the spectral tuning curves of cortical neurons, but this effect was dependent on the pairing frequency. Specifically, if the pairing frequency was above the BF, BFs shifted toward lower frequencies, i.e. away from the pairing frequency. If the pairing frequency was below the BF, no significant changes occurred. In addition, tPA increased the tuning width, i.e. tuning curves were wider following pairing in the presence of tPA. Finally, tPA resulted in reduced neuronal gains following pairing. These data show that tPA influences synaptic plasticity and tuning curves in the adult auditory cortex, in a frequency pairing paradigm. To the best of our knowledge, it is the first study to show that tPA affects neural tuning characteristics *in vivo.* We will first discuss our results in light of previous pairing protocols, followed by a discussion of potential mechanisms by which tPA could promote these changes.

While we also found changes following pairing when saline was applied, these were more limited and critically differed from those seen when tPA was applied. Overall, they were more reminiscent of changes reported as “sensitization”-related changes in previous studies.^42^ That is, the repeated presentation of the pairing stimulus on its own (without concurrent unconditioned stimulus presentation, drug application, or stimulation of neuromodulatory nuclei) resulted in an increase in tuning gain (height of the tuning function) when pairing was done with a stimulus above the BF and a concurrent increase in tuning width (FWHM). This is reminiscent of the frequency-independent increase in activity following “sensitization” that has been reported.^3^^,^^16^^,^^42^ More pronounced and more specific changes to, for example, BF tuning were restricted to tPA-applied conditions.

### Auditory spectral tuning and its modification by pairing protocols

The effects seen in our study differ from those seen when classical conditioning paradigms were used. These paradigms employed aversive (fear) conditioning, whereby a conditioned stimulus (CS+, a specific tone frequency) was paired with an unconditioned stimulus (US), a foot or leg shock,.^7^^,^^42^^,^^43^ Classical conditioning resulted in tuning curve shifts toward the CS+ frequency, irrespective of the location of the CS+ tone relative to the original BF. These changes were usually induced by increased responses to the CS+ frequency and reduced responses to the original BF. Similar results could be obtained by electrical stimulation of the basal forebrain cholinergic system.^3^^,^^44^^,^^45^^,^^46^^,^^47^ Here, electrical stimulation of the basal forebrain, paired with a specific auditory stimulus, resulted in altered spectral tuning curves, whereby the paired frequency became more strongly represented.

While these results point to roles of acetylcholine (ACh) in inducing adult neural plasticity, the results from local application of ACh in conjunction with frequency pairing were somewhat more complex. Localized iontophoretic application of ACh in anesthetized cats often caused pronounced response attenuation to the paired frequency, sometimes paired with increased responses to flanking frequencies.^48^^,^^49^ The former could cause some tuning curve shifts away from the paired frequency, while the latter would result in increased tuning bandwidth. Both effects are reminiscent of those seen with our protocol. In addition, in some cases non-frequency-selective changes occurred in the form of general response enhancement or reduction.^49^ In bats, BF shifts induced by stimulus conditioning were augmented by ACh^7^ and NMDA^45^ application.

Electrical stimulation of the locus coeruleus noradrenergic system^16^ could also alter auditory tuning curves, and while these could resemble BF shifts toward the pairing frequency, there were also frequency-selective reductions in response rate following the pairing. Hence, systematic tuning shifts as seen with basal forebrain stimulation were not encountered at cortical level after locus coeruleus stimulation. Local iontophoresis of noradrenaline combined with frequency pairing generally caused frequency-specific reduction of responses, i.e. the pairing frequency resulted in lower responses after the pairing.^15^

Our data of BF shifts away from the pairing frequency are therefore somewhat more reminiscent of noradrenaline-induced effects, but it is important to point out that we did not encounter the highly specific response reductions described in some of the studies.^15^ Importantly, the effects of locally applied ACh or noradrenaline differed from effects encountered by electrical microstimulation of neuromodulatory brain stem nuclei. The former^15^^,^^48^^,^^49^ resulted in effects somewhat reminiscent to the local application of tPA that was used in our study. This suggests that the highly specific CS+ retuning seen with behavioral or neuromodulatory brain stem microstimulation is mediated through more complex interactions, involving cortex (brain)-wide effects,^50^^,^^51^ which alter cortico-cortical interactions^52^^,^^53^^,^^54^ and also recruit GABAergic and glutamatergic drive in the case of basal forebrain stimulation.^55^^,^^56^

### Possible mechanistic effects of tPA

How could tPA cause the plasticity seen in our study? Many insights into the role of tPA in relation to neuronal function come from *in vitro* studies, where it affects different forms of synaptic plasticity.^25^^,^^28^^,^^29^^,^^31^^,^^57^^,^^58^ Late-phase long-term potentiation (L-LTP) and synaptic growth in hippocampal mossy fiber pathway are increased by exogenous tPA (added to hippocampal slice cultures) and in mice overexpressing tPA.^28^^,^^29^ Behaviorally, this leads to improved spatial learning. Conversely, studies using tPA gene knockout mice show defects in LTP and long-term depression (LTD) in the striatum^59^ and LTP in hippocampus.^31^

tPA can be released from cortical neurons, where it triggers structural and functional changes to presynaptic and postsynaptic densities^60^ and associated changes in synaptic efficacy. The latter is achieved by increased transmitter release and increased recruitment of AMPA and NMDA receptors into the postsynaptic density.^34^^,^^61^ tPA can also modulate NMDA receptor function via its capacity to bind to NMDA receptor subunits.^32^^,^^62^^,^^63^ These mechanisms should result in a shift of the BF toward the pairing frequency, not the repulsion seen in our study. However, the mechanisms of AMPA recruitment to the postsynaptic density, mediated through Ca + NMDA influx and pCaMKIIα activation, is dependent on baseline pCaMKIIα.^34^ AMPA receptor recruitment only happens with low levels of baseline pCaMKIIα. If baseline pCaMKIIα levels are high, it results in removal of AMPA receptors from the postsynaptic density and hence a possible reduction of responses to the pairing frequency stimulus. It might thus be the case that in our preparation the baseline levels of pCaMKIIα were relatively high.

Mechanisms by which tPA could reduce synaptic efficacy is by inhibition of NMDA receptor-mediated increases in intracellular calcium^64^ or NMDA receptor subunit cleavage.^32^^,^^33^^,^^65^ Such changes would result in a reduced response to the pairing frequency, associated with overall shifts in BF.

tPA also results in reduced numbers of NMDA receptor subtype 2B (NR2B) signaling through endocytosis of NMDA receptors containing NR2B subunits.^66^ NR2B subunits are possibly more expressed on inhibitory interneurons,^67^ and hence tPA could lead to reduced inhibition. Inhibition is a critical component in shaping spectral tuning curves. It has been suggested that inhibition is broad toward high frequencies (relative to the BF), resulting in steep high-frequency side cutoffs of the tuning curve, creating so called “slant-lower units”.^68^^,^^69^ In addition, inhibition contributes to response normalization.^70^^,^^71^ Reduced inhibition might cause the widening of tuning functions and the increased gain seen for higher-pairing conditions. However, reduced inhibition of the above-mentioned steep high-frequency side cutoffs would result in tuning shifts toward higher-pairing stimuli, not away from them. If anything, the repulsion might be a sign of increased inhibition with higher pairing. Furthermore, tPA application resulted in reduced gains following pairing, not increased gains. So, altered inhibition by itself is unlikely to account for the different phenomena encountered in our study.

### Conclusion and outlook

Our study demonstrates that tPA affects neural tuning function in adult mouse cortex in multiple ways. It raises the prospect that perceptual learning abilities might be affected by neuronal tPA manipulation,^72^^,^^73^^,^^74^^,^^75^ and that tPA^−/−^ knockout mice might have an altered auditory phenotype. Rather than injection of exogenous tPA, the same protocol could be used in mice with higher levels of endogenous tPA compared with normal mice. This eliminates the problem of limited half-life of recombinant tPA. Similarly, a comparison could be made between tPA^−/−^ mice and tPA^+/+^ mice undergoing the same pairing protocol. Whether tPA was implementing these effects directly or via plasmin formation cannot be determined from our studies and will require further investigations, for example using direct plasmin inhibitors with tPA or conducting similar experiments in plasminogen-deficient mice.

### Limitations of the study

While we demonstrate that tPA can affect neuronal plasticity in the mouse auditory cortex, our study does not reveal the underlying mechanisms. For example, we are not in a position to know if this effect of tPA required its catalytic effect or whether plasmin generation was necessary. Using a non-proteolytic form of tPA (i.e., with the active serine replaced) or tPA-inhibitor complexes that have been previously reported to alter blood-brain permeabililty in mice (Sashindranath et al., 2012) or using mice deficient in either tPA or plasminogen would enable us to pinpoint these mechanisms more precisely. Moreover, tPA−/− mice would provide the opportunity to perform rescue experiments and to also see if tPA-deficient animals present a defect of tuning curves. Similarly, mice that neuronally overexpress tPA^29^ would also allow us to determine the effect of increasing levels of endogenous tPA on auditory plasticity. Additional studies are hence required to explore these various possibilities.

## STAR★Methods

### Key resources table

**Table:** 

REAGENT or RESOURCE	SOURCE	IDENTIFIER
**Chemicals, peptides, and recombinant proteins**		

tissue type plasminogen activator (tPA)	(Alteplase", Boehringer Ingelheim, Germany) was obtained from expired stocks from the pharmacy of the Alfred Hospital	NA

**Deposited data**		

data	gitlab	https://gitlab.com/alex2thiele/tpa/-/blob/main/zscore_fit https://gitlab.com/alex2thiele/tpa/-/blob/main/norm_fit
data analysis software	gitlab	https://gitlab.com/alex2thiele/tpa/-/blob/main/zscore_fit https://gitlab.com/alex2thiele/tpa/-/blob/main/norm_fit

**Experimental models: Organisms/strains**		

C57BL/6 adult mice	Charles River UK	NA

**Software and algorithms**		

data analysis software	MATLAB (2017,2018,2021) Mathworks	http://mathworks.com
data acquisition software	Cheetah (Neuralynx)	5.6.3.0

**Other**		

iBOND Total Etch	Heraeus Kulzer	NA
Charisma	Heraeus Kulzer	NA
Tetric EvoFlow	Ivoclar Vivadent	NA
micropipettes	Brand Blaubrand IntraMARK	NA
laminar electrodes	ATLAS electrodes	E16R-50-S1-L10

### Resource availability

#### Lead contact

Further information and requests for resources should be directed to the Lead Contact, Alexander Thiele (alex.thiele@ncl.ac.uk).

#### Materials availability

This study did not generate new unique reagents.

### Experimental model and subject details

#### Animals and procedures

Female C57BL/6 adult mice (aged 2-7 months) were used for this study. We performed between 3 and 13 experiments per animal. The time interval was at least 24 h between tPA injections. In general, the time interval between tPA vs. control (saline) injections was at least 48 h (except for 2 recording sessions, where saline recordings were performed 24 h after a tPA injection).

All procedures were compliant with the European Communities Council Directive RL 2010/63/EC, the US National Institutes of Health Guidelines for the Care and Use of Animals for Experimental Procedures, and the UK Animals Scientific Procedures Act. Ethics approval was obtained from Newcastle (Animal Welfare Ethical Review Board) and Monash (Alfred Medical Research Ethics Precinct) Universities ethics committees and was covered by a UK project licence.

### Method details

For initial surgeries, anesthesia was induced with 5% isoflurane gas and maintained with 2% isoflurane. Surgery commenced with a craniocaudal incision along the scalp. The skull was exposed by blunt dissection, separating the skin from underlying connective tissue and detaching the temporalis muscle. The location of the primary auditory cortex (A1) was marked on the skull, based on stereotactic coordinates at between 2.4 and 2.8 mm (mm) posterior and 4.0-4.1 mm lateral to bregma. Total etch dental bonding acrylic (iBOND Total Etch, Heraeus Kulzer) was then applied to the bone surface and set with UV light. Charisma ceramic dental bonding acrylic (Charisma, Heraeus Kulzer) was then applied to the skin-skull margin. Tetric EvoFlow (Ivoclar Vivadent) was used to fix a custom-made stainless-steel chamber over the pre-marked site of the auditory cortex as well as a head-holder on the posterior skull aspect. Curing was achieved using UV-light.

Pre-operatively, subcutaneous meloxicam (5 mg/kg) (with or without buprenorphine (0.1 mg/kg) depending on the operator) were administered. Daily doses of subcutaneous meloxicam (5 mg/kg) were administered for 2 days post-operatively. The animal was monitored until it was awake from anesthesia and moving independently. Post-operative checks took place at one and 3 h after surgery. The cage was transferred to an incubator for one night prior to the animal returning to its home cage.

#### Auditory stimuli and experimental hardware

All experiments were conducted inside a sound-proof box, external diameter ∼1200 mm (H) x 924m (L) x 900 mm (W) layered internally with Rockwool Pro Rox (SL960) soundproof material) and with an additional inner lining consisting of a 55 mm thick pyramidal melamine sound proof foam (F470 Pyramid Mela). The entire assembly was placed inside a Faraday Cage.

Auditory stimuli were created using MATLAB. These were pure tones of 300 ms length with a 50ms on and off ramp. The frequencies used ranged from 1 kHz to 32 kHz, whereby each successive frequency was ∼1.1892 times the previous frequency (i.e. ∼1/4 octave steps). This ensured that we presented 21 frequencies in total, including 1 kHz, 2 kHz, 4 kHz, 8 kHz, 16 kHz and 32 kHz exactly, with 3 interspersed frequencies between each step. These stimuli are within the normal mouse hearing range 1 kHz–100 kHz,.^76^

We used CORTEX software (Laboratory of Neuropsychology, National Institute of Mental Health, http://dally.nimh.nih.gov/index.html) run on a computer with an Intel Core i3-540 processor to present each auditory stimulus via a Microsoft Direct X script. Computer output was transmitted via a Creative Technologies SoundBlaster Audigy FX PCle 5.1 SB1570 audio card to a Cyrus 8DAC amplifier and ELAC BS 403 loudspeakers. To ensure that accurate sound intensities were delivered, all stimuli were calibrated using a ⅛ inch pressure-field microphone and preamplifier (Brüel + Kjær type 4138-C-006 and type 2669) connected to a measuring amplifier (Brüel + Kjær type 2636) and digital oscilloscope (Rhode + Schwarz HM03002). For calibration, the microphone was placed inside the soundproof box at the position, where the mouse’s head would be during the experiment.

Figure S1 gives an overview of the experimental and presentation procedure. Stimuli were presented at 4 different intensity levels, namely 50, 60, 70 and 80 dB sound pressure level [SPL]. This allowed characterisation of the frequency tuning of each unit. To determine tuning curves, stimuli were presented pseudo-randomly at each intensity (84 stimuli total) with 1.8 s interstimulus intervals between each tone. Following presentation of the 84 stimuli, the procedure was repeated 10 times in total, resulting in 10 repetitions for each of the 84 stimuli. Neuronal activity following stimulus delivery was displayed online using a MATLAB R2014a (MathWorks) custom written script. Neural responses were converted to z-scores

$$zscore=\frac{mean\left(stimulus\;evoked\;activity\right)-mean\left(spontaneous\;activity\right)}{standard\;deviation\left(spontaneous\;activity\right)}$$

relative to interstimulus interval activity, and color coded for online assessment of neural tuning curves (Figure 1 as examples). Based on these tuning curves the conditioning stimuli were chosen to be either at the lower flanking edge of the tuning curve, or at the upper flanking edge of the tuning curve. Presenting conditioning stimuli at the lower and the upper flanking edge was done in line with previous pairing studies e.g.,^15^ and to counterbalance the possibility that conditioning effects differ depending on the placement relative to the BF. We aimed to place the conditioning frequency within ∼0.5 of an octave of the estimated BF. Within an experimental session this was done based on visual inspection of the multi-unit online recorded activity. On average the conditioning frequency was within the intended range of the BF. However, BFs assessed after spike sorting and after offline analysis (with BF quantification by means of fitting tuning functions with a Gaussian) sometimes differed from online assessment. Hence the conditioned frequency could occasionally be closer to the BF than 0.5 octaves difference, or further from the BF than 0.5 octaves. Overall the median difference was 0.41 octaves, the mean was 0.50 (+/−0.38 std) octaves difference, and the 20/80 percentile range was 0.13–0.84 octaves difference.

Conditioning was performed by the presentation of the conditioned frequency 30 times (at 80 dB[SPL]) for 300ms with 50ms on and off ramps, with a 30 s interstimulus interval. Following conditioning, another tuning-curve was obtained immediately afterward, using the protocol described above.

Each mouse on any given day had an equal chance of experiencing one of four experimental conditions (control vs. drug treatment, pairing with a conditioning frequency above or below the best frequency, Figure S1 for details), to avoid potential order or bias effect.

#### Craniotomy

A craniotomy was made under anesthesia inside the mouse’s chamber using a dental drill, at the location described above. This craniotomy was used in all subsequent experiments.

#### Substance delivery

Pipettes used to inject experimental substances to the brain, were created by pulling 5 μL glass micropipettes (Brand Blaubrand IntraMARK) in a glass micropipette puller (Narishige type PE-21). Marks were made at 1 mm intervals along the length the pulled micropipettes using a fine-tipped permanent marker to represent 70 nL increments.

Each intracranial injection delivered the chosen substances according to Table S1. The ‘amount required’ differs from the ‘amount delivered’, as substances could only be injected accurately in ∼70 nL increments. Pipettes were stereotaxically inserted into A1 and substances were injected at depths of 1.5 mm, 1.0 mm and 0.5 mm along a single injection tract. Prior to use, substances (except saline) were stored at temperatures between −75 and −85°C.

#### Electrode placement and insertion

Following substance delivery, the electrode was positioned as close to the injection site as possible and was deemed to be a depth zero (0 mm), when it was just touching the cerebral cortex. Electrodes were inserted using a Narishige MO-95 microdrive. We used E16R-50-S1-L10 ATLAS laminar electrodes PCB (Omnetics) for all recordings. Electrodes were inserted until spikes could be seen on the connected Cheetah 5.6.3.0 (Neuralynx) acquisition system on at least the first 3-5 channels. This occurred after the electrode had been inserted approximately 200-350 μm from its initial position.

#### Data acquisition and spike extraction

Details have been published previously^77^ and are copied here for ease of access. Neuronal data were collected by Cheetah data acquisition (Neuralynx) interlinked with Remote Cortex. Raw data were acquired at a sampling frequency of 32,556 Hz with a 24-bit analog-to-digital converter, with minimum and maximum input ranges of 11 and 136,986 μV respectively (pre-set by Neuralynx, Inc.), a DMA buffer count of 128, and a DMA buffer size of 10 ms, using a 64-channel Digital Lynx 16SX Data Acquisition System (Neuralynx, Inc.). Digital referencing of voltage signals was performed prior to the recording of raw data, using commercially provided Cheetah 5 Data Acquisition Software v. 5.4.0 (Neuralynx, Inc.).

Following each recording session, the raw data were processed offline using both commercial (Neuralynx, Inc.) and custom-written (MATLAB, Mathworks) software. Signals were extracted using Cheetah 5 Data Acquisition Software. The sampling frequency remained the same (32,556 Hz), while the input range settings were individually tailored to each session, with band-pass filter frequency set to a low cut frequency of 600 Hz and a high cut frequency of 9000 Hz, and saved at 16-bit resolution. Following extraction of thresholded spike waveforms, unit action potentials were extracted manually using Neuralynx Spikesort 3D v2.5 software. We included single and multi-units into the analysis for this paper. We refer to both as 'units' throughout the paper and do not distinguish between them.

### Quantification and statistical analysis

#### Population

The data used for analysis was recorded from 27 individual experiments. These were recorded across 9 different mice. We recorded 42 cells with pre- and post-conditioning tuning curves when saline was injected and 44 cells when tPA was injected.

#### Parameters of analysis

The spikes elicited from units in response to each auditory stimulus were extracted from a 200 ms response window from 70 to 270 ms after stimulus onset, accounting for initial stimulus on ramp (50ms) and response latency.

#### Normalisation and Gaussian fit

Neuronal spike data were normalised for each unit in a recording session by counting spikes in the above mentioned response window, and normalizing all other responses relative to the one with the highest count.

Additionally, we calculated z-scored activities from average single trial spiking activity whereby the stimulus induced z-scored activity (for a given frequency and intensity) was:

$${z-score}_{condition}=\frac{{mean\;activity}_{condition}-{mean}_{spontaneous}}{{standard\;deviation}_{spontaneous}}$$

Neuronal BFs were determined from the normalised data, and from the z-scored data by first averaging across the 4 stimulus intensities and then selecting the frequency which elicited the overall maximum neuronal response.

In addition, BF, tuning width, amplitude and offset were determined by fitting normalized spike data and z-scored spike data using a Gaussian function,

$$g\left(x\right)=\frac{1}{\sigma \sqrt{2\pi }}e^{-{\frac{1}{2}\left(\frac{x-\mu }{\sigma }\right)}^{2}}$$

Here the average across all 4 stimulus intensities was used as dependent variables, while the frequencies constituted the independent variable. These fits yielded 4 parameter estimates of interest to our study, namely the location of the BF (peak location of the Gaussian tuning curve in the frequency domain), the tuning width, expressed as full width at half maximum of the tuning curve (FWHM), the height of the tuning curve at peak location relative to its minimum (Rmax, also referred to as gain in the remainder of the manuscript), and the offset of the tuning curve (offset, i.e. the minimum of the tuning curve). Fitting was done using the MATLAB 2020a fminsearch function. Fitting was constrained such that (A) the BF had to be within the range of frequencies tested, (B) the tuning width (in octaves) had to exceed 0.0235, (C) the maximum of the tuning function could not be larger than 1.5 times the overall measured maximum activity (normalized or z-scored), and (D) the tuning minimum had to be larger than 0 when fitting was done to normalized data (spiking activity cannot be negative). As z-scored data can be negative, we constrained the offset not to deviate by more than 50% from the minimum z-scored activity.

To assess the quality of fits we calculated the variance accounted for as previously published.^78^ We included cells into our population sample if the fit accounted for at least 70% of the variance in the pre-pairing and in the post-pairing data (but see controls where we relaxed this requirement).

Effects of different types of manipulation on parameters of the neuronal tuning curves were investigated using a mixed-model ANOVA(MATLAB 2020a, using the fitglme function followed by an ANOVA), and results are reported as F-statistics, and effect sizes in the form of explained variance (η^2^_p_).^79^ All (post-hoc) pairwise comparisons were based on two-sided tests and are reported as t-statistics, and as z-statistics for non-parametric tests. Effect sizes are reported, along with confidence intervals (CI). Pairwise test effects sizes are reported as Cohen’SD for independent measures and Cohen’s D_z_ for repeated measures.^79^ Correction for multiple comparisons, where appropriate, was done based on false discovery rate correction FDR,.^80^

In addition to the model based assessment of change in tuning parameters (the fitted functions), we determined the difference in measured activity peak location and height (averaged across all 4 stimulus intensities) between pre- and post-pairing using a t-test. A systematic change toward higher or lower frequencies would result in a significant difference.

#### Injection analysis using *in situ* zymography

To confirm that the injected tPA had been correctly targeted to the auditory cortex, *in situ* zymography was undertaken These experiments were conducted on mice with cranial implants once electrophysiological recordings had concluded. 420 nL of human tPA (66 μM) and HEPES buffer was injected into the auditory cortex, using the same coordinates and procedures used for the intracranial injections during previous experiments.

Once injection was complete, a ketamine/medetomidine anesthetic combination was given prior to tissue collection and the mouse was transcardially perfused with normal saline to remove circulating blood from the brain.

7 days post-freezing, injected brains were packaged and sent from the UK to Australia where *in situ* zymographic analysis of brain sections provided insight into the location and spread of the injected tPA post-injection. Briefly, brains were sectioned (100 μm) using a cryostat and placed onto a Super Frost Plus slide. An overlay mixture (10 mM Tris-HCl, pH 7.4, 1% (w/v) low-melt agarose, 4% boiled skim milk, 0.1% Triton X-100 and 12.5 μg/ml plasminogen) was prepared at 50°C. 400 μL of mixture was pipetted evenly onto pre-warmed slides and glass coverslips were placed on top. Slides were incubated for up to 6h in a humidified 37°C incubator. Images were captured using a scanner.

## Data Availability

• Data necessary to reproduce the figures have been deposited at Database: https://gitlab.com/alex2thiele/tpa/-/blob/main/zscore_fit and at Database: https://gitlab.com/alex2thiele/tpa/-/blob/main/norm_fit and are publicly available as of the date of publication. DOIs are listed in the key resources table. • Code necessary to reproduce the figures has been deposited at Database: https://gitlab.com/alex2thiele/tpa/-/blob/main/zscore_fit and at Database: https://gitlab.com/alex2thiele/tpa/-/blob/main/norm_fit and is publicly available as of the date of publication. DOIs are listed in the key resources table. • Any additional information required to reanalyze the data reported in this paper is available from the lead contact upon request.
