# Supplementary material for: Tissue-type plasminogen activator induces conditioned receptive field plasticity in the mouse auditory cortex
Source: iScience. 2023 Jan 7;26(2):105947. doi: 10.1016/j.isci.2023.105947 (PMC9874071; doi:10.1016/j.isci.2023.105947)
Supplement: Document S1. Figure S1 and Table S1 [file mmc1.pdf]

iScience, Volume *26*

Supplemental information

Tissue-type plasminogen activator induces conditioned receptive field plasticity in the mouse auditory cortex

Caitlin Smart, Anna Mitchell, Fiona McCutcheon, Robert L. Medcalf, and Alexander Thiele

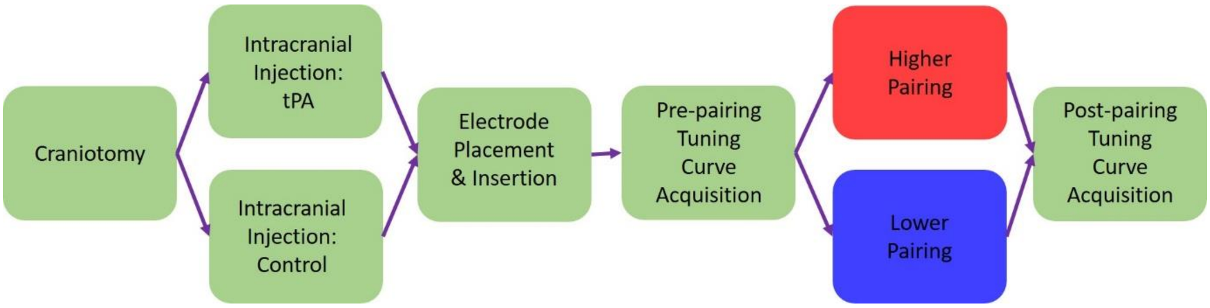

Supplemental Figure S1: Experimental protocol. Related to STAR Methods. The initial phase of the experimental protocol was completed under anaesthesia, while stages following electrode placement were completed while the mouse was awake (i.e. it had recovered from anaesthesia). Each mouse on any given day had an equal chance of experiencing one of four experimental conditions, namely tPA or saline injection, and higher or lower pairing. Unsystematic, but balanced assignment of these conditions in each mouse minimized order effects and bias.

Substance	Concentration	Amount Required	Amount Delivered
**Human tPA**	58μM	261nl (1μg)	280nl
	66μM	229nl (1μg)	210nl
**Saline**		261nl	280nl
		522nl	560nl

Supplementary Table T1: Substances and quantities used. Related to STAR Methods. Biologically active and control substances used as the conditioned stimuli in the experimental protocol.
